# Gene Expression-Based Biomarkers for *Anopheles gambiae* Age Grading

**DOI:** 10.1371/journal.pone.0069439

**Published:** 2013-07-23

**Authors:** Mei-Hui Wang, Osvaldo Marinotti, Daibin Zhong, Anthony A. James, Edward Walker, Tom Guda, Eliningaya J. Kweka, John Githure, Guiyun Yan

**Affiliations:** 1 Program in Public Health, University of California Irvine, Irvine, California, United States of America; 2 Chao Family Comprehensive Cancer Center, UCI Medical Center, Orange, California, United States of America; 3 Department of Molecular Biology & Biochemistry, University of California Irvine, Irvine, California, United States of America; 4 Department of Microbiology & Molecular Genetics, University of California Irvine, Irvine, California, United States of America; 5 Department of Microbiology and Molecular Genetics, Michigan State University, East Lansing, Michigan, United States of America; 6 Human Health Division, International Centre of Insect Physiology and Ecology (ICIPE), Nairobi, Kenya; 7 Centre for Global Health Research, Kenya Medical Research Institute, Kisumu, Kenya; 8 Division of Livestock and Human Diseases Vector Control, Tropical Pesticides Research Institute, Ngaramtoni, Arusha, Tanzania; Quensland University of Technology, Australia

## Abstract

Information on population age structure of mosquitoes under natural conditions is fundamental to the understanding of vectorial capacity and crucial for assessing the impact of vector control measures on malaria transmission. Transcriptional profiling has been proposed as a method for predicting mosquito age for *Aedes* and *Anopheles* mosquitoes, however, whether this new method is adequate for natural conditions is unknown. This study tests the applicability of transcriptional profiling for age-grading of *Anopheles gambiae*, the most important malaria vector in Africa. The transcript abundance of two *An. gambiae* genes, AGAP009551 and AGAP011615, was measured during aging under laboratory and field conditions in three mosquito strains. Age-dependent monotonic changes in transcript levels were observed in all strains evaluated. These genes were validated as age-grading biomarkers using the mark, release and recapture (MRR) method. The MRR method determined a good correspondence between actual and predicted age, and thus demonstrated the value of age classifications derived from the transcriptional profiling of these two genes. The technique was used to establish the age structure of mosquito populations from two malaria-endemic areas in western Kenya. The population age structure determined by the transcriptional profiling method was consistent with that based on mosquito parity. This study demonstrates that the transcription profiling method based on two genes is valuable for age determination of natural mosquitoes, providing a new approach for determining a key life history trait of malaria vectors.

## Introduction

*Anopheles* mosquitoes transmit malaria, causing more than 247 million clinical cases and killing about ∼655,000 people annually, with more than 90% malaria-related deaths in Africa [1]. Female mosquitoes become infected with malaria parasites by ingesting a blood meal from an infected person and subsequently transmit the pathogens when biting an uninfected human host. However, female mosquitoes only transmit parasites after the ingested *Plasmodium spp* gametocytes undergo a series of developmental changes that result in the production of infective salivary gland sporozoites. The interval between the acquisition of parasites by mosquitoes and the maturation of infectious stages, termed as the extrinsic incubation period (EIP), is dependent on environmental temperature and usually ranges from 9–14 days [2]. Only those female mosquitoes that survive longer than the EIP are capable of transmitting malaria [3].

New vector-borne diseases control programs aiming at shifting the age structure of the mosquito population toward younger age classes incapable of transmitting pathogens have been proposed. For example, insecticides with a delayed rather than immediate killing effect have been proposed as a strategy to target primarily older infectious mosquitoes and interrupt transmission while delaying the development of insecticide resistance in malaria vectors [4]. *Wolbachia* bacteria that reduce mosquito reproductive success and longevity have been transferred successfully into malaria mosquitoes [5], [6]. More traditional malaria control measures such as insecticide-treated bed nets not only reduce malaria vector abundance, but also likely reduce the life span of mosquitoes [7], [8]. In addition to malaria control measures, environmental factors affect mosquito development [9], [10] and climate change is expected to alter mosquito survivorship [11]. Hence, information on the age structure of *Anopheles* mosquitoes is essential to assess the impact of environmental and climatic changes and vector control measures on malaria transmission.

Existing mosquito age-grading methods based on morphological changes in the reproductive system [12], [13] or cuticular hydrocarbons [14], [15] lack sufficient reliability and sensitivity. Transcriptional profiling has been tested as an alternative for mosquito age grading in *Aedes* mosquitoes [16], [17], however, there is no study on the suitability of this new technique for field populations. We examined two *Anopheles gambiae* genes identified previously from microarray studies [9] that exhibit monotonic age-dependent changes in transcript accumulation levels to test further the applicability of a transcriptional profiling method for mosquito age grading. These potential biomarkers were used to evaluate mosquitoes maintained either in semi-natural environments or derived from the wild. The use of transcriptional profiles based on limited number of genes provides an important new tool for understanding malaria transmission and evaluating the impact of malaria vector control, and helps to solve the longstanding problem of difficulty in determining the age of field-caught mosquitoes.

## Materials and Methods

### 1. Mosquito Populations

#### Laboratory cage populations

Two laboratory *An. gambiae* strains were used in the experiments to determine the correlation between transcription profiles and mosquito age. Each strain was maintained under different environmental conditions. The G3 strain was obtained from the Malaria Research and Reference Reagent Resource Center (MR4 Manassas, VA, USA) and the colony was reared at the University of California, Irvine (UCI), in an insectary under tightly-regulated conditions of constant temperature and humidity. The Mbita strain originated from Mbita Point, Suba District in western Kenya and has been maintained since 1999 [18] in the Mbita Point Field Station, International Centre of Insect Physiology and Ecology (ICIPE), Kenya, in an insectary lacking regulated conditions for temperature and humidity. Mosquitoes at UCI were reared at a relative humidity of 75%, temperature of 26°C, and 12∶12 hour light-dark cycle. Mosquitoes at Mbita were reared at an unregulated humidity of 61±7%, temperature of 27±2°C, and 12∶12 hour light-dark cycle. Mosquito larvae were reared in trays of 150 first-instar larvae per liter of water, and fed with a mixture of Tetramin® (Tetra Werke, Melle, Germany) fish food and yeast. Adult mosquitoes were maintained in cages with 250 mosquitoes per cage volume of 128 oz (with 18 cm diameter and 21 cm height), with an equal sex ratio. Adults had access to raisins and cotton balls saturated with deionized water (UCI lab condition) or 6% sugar water (Kenya). Overall, the mosquitoes were maintained in optimal conditions as described previously [9], [19]. Anesthetized rabbits were used for blood feeding every 4 days. Mosquitoes, blood fed or not, were randomly collected every 5 days from day 1 to day 30 post emergence. All mosquitoes were preserved in RNAlater® (Sigma) and maintained at −20°C before RNA extraction.

#### Mosquitoes in MalariaSphere

A hybrid mosquito population of the Mbita and Kisumu strains [20] was used to determine the general applicability of the age-grading biomarkers. The population was maintained in the MalariaSphere in ICIPE’s Mbita Point Field Station. MalariaSphere is a simulated ecosystem for semi-field studies of Anopheline mosquitoes that contains near-natural breeding sites, a local traditional-style house, and different types of food crops and indigenous wild plants to mimic the natural environment of adult *An. gambiae* [21]. The temperature and relative humidity inside MalariaSphere is comparable to the surrounding natural ambient conditions. *Anopheles gambiae* held in MalariaSphere can complete their entire life cycle and participate in all the major life-history behaviors (mating, sugar feeding, oviposition and bloodfeeding) within the enclosure [21]. Five-hundred each freshly-emerged female and male *An. gambiae* mosquitoes were released into the MalariaSphere, but only female mosquitoes were collected for age grading. Access to human volunteers sleeping within the MalariaSphere was provided twice a week during the course of the study. Ten to fifteen mosquitoes resting in the huts of the MalariaSphere were collected every 5 days until no female adults were available for collection. All mosquitoes were preserved in RNAlater and maintained at −20°C before RNA extraction.

#### Mosquitoes used for Mark-Release-Recapture (MRR) experiments

MRR experiments were performed in the MalariaSphere to determine the validity of the age-grading biomarkers against mosquitoes of known age. HOBO data loggers were placed inside at beginning to monitor hourly temperature changes. Five groups (1, 5, 10, 15 and 20 days post emergence) of female *An. gambiae* mosquitoes of Mbita strain were reared, transferred in batches of 50 individuals to small paper cups, immobilized by cold and dusted gently with fluorescent powder (Day-Glo Color Co., Cleveland OH, USA). The chronological age for different age groups of mosquitoes were converted into degree days based on the real time data from HOBO devices Mosquitoes of each age group were marked with one of five fluorescent powders (green, yellow, pink, blue and dark-red). Fluorescent-marked mosquitoes recovered from cold-shock were provided access to 6% sucrose solution in cotton balls. After a 24 hour recovery period, 600 marked female mosquitoes from each age group were released into one MalariaSphere. About 20 mosquitoes were re-captured randomly each day, and a total of 151 mosquitoes among the 3,000 marked and released mosquitoes were recaptured during the 8 day post-release period. The mosquitoes were collected inside the hut, a traditional Kenyan homestead, located in the MalariaSphere and human volunteers sleeping in it every other day. The fluorescent color was diagnosed with an ultraviolet light (UV) LED flashlight immediately after re-capture and confirmed at by UV transilluminator with increased UV intensity and uniformity. Mosquitoes were preserved in RNAlater singly and maintained in −20°C for RNA extraction.

#### Field-collected mosquitoes

Mosquitoes were collected from Mbita Point (34°12′E, 0°26′S), Suba district and Kisian (34°75′E, 0°10′S), Kisumu District, Western Kenya in April 2010 to determine age structure of natural mosquito populations. Both sites lies on the shore of Lake Victoria, and the rainy season generally starts in late April and ends in June. *Anopheline* mosquitoes were collected indoor in the morning using the pyrethrum spray catch method [22], [23]. The knockdown mosquitoes were preserved in RNALater into a sterile 2 ml storage tube immediately in field. The samples were transported immediately to field laboratory. A total of 41 female *Anopheles* mosquitoes from Mbita Point were dissected to determine the parity status [24], and then stored in RNAlater individually for subsequent species identification [25] and RNA extraction. The hourly temperature during the sampling period was obtained for each site. Female mosquitoes from the Kisian site were not dissected. A total of 71 *An. gambiae* female mosquitoes from Kisian were examined for their transcription profiles.

### 2. Total RNA Extraction and Transcript Preparation

Total RNA from individual mosquitoes was extracted using a QIAGEN RNeasy® Mini kit (QIAGEN Inc., Valencia, CA). Samples were removed from RNAlater and transferred to 1.5 ml microfuge tube, homogenized with 350 µl of lysis buffer with β-mercaptoethanol following the manufacturer’s instructions. Total RNA was collected in 30–50 µl RNAse-free water and concentrations determined by absorbance readings using a Nanodrop ND-1000 spectrophotometer (Thermo Scientific, USA). RNA samples were stored at −80°C. Reverse transcription reactions were performed with 400 ng of DNAse-treated RNA using QIAGEN QuantiTect® Reverse Transcription reagents following the manufacturer’s protocols.

### 3. Quantitative Gene Amplification with Age-grading Molecular Markers

In a previous study we identified 7 genes showing monotonic decreasing or increasing expression pattern with *An. gambiae* aging in the G3 strain, and these were not affected by blood feeding or insecticide resistance [9]. Two genes (AGAP009551 and AGAP011615) exhibited consistent decreasing expression with mosquito age across mosquito strains and climate conditions. Therefore, these two genes were selected as the candidate age-grading biomarkers for field evaluation in Kenya. Quantitative gene amplification (qRT-PCR) was conducted as described previously [9]. Primers and Zen double-quenched probe (Integrated DNA Technologies) were designed for these genes using Primer 3.0 (http://www.broad.mit.edu/cgi-bin/primer/primer3_www.cgi) (Table 1). The ZEN double-quenched probe has an internal ZEN quencher nine nucleotides from the 5′ fluorophore to increase the accuracy and reliability of 5′ nuclease qRT-PCR experiments. Real-time quantifications were performed using the total RNA samples isolated from individual mosquitoes, with the 2X PCR Master Mix (QIAGEN) on a MJ Research DNA Engine Opticon RT-PCR System (Bio-Rad). The running program was set to 95°C, 3 min, followed by 44 cycles of 95°C for 30 s, 60°C for 30 s, and 72°C for 1 min, and a final step of 72°C for 10 min. All qRT-PCR assays were run in triplicate. Ct values were calculated as the second derivative maximum of the fluorescence curve using the comparative quantification analysis module in the MJ Research Opticon RT-PCR System (MJ OpticonMonitor™, version 3.1). The transcript abundance of AGAP009551and AGAP011615 was normalized against the transcript abundance of the *An. gambiae* ribosomal protein S7 encoding gene (AGAP009613) by calculating Δ-Ct values for each gene in each mosquito. We used the same amount of total RNA when preparing the cDNA. For each single sample (mosquito), we ran at least 3 times of qRT-PCR on both S7 and target age-grading genes respectively. The delta CT values of S7 for each sample were either the same or very close (±1) within the same population.

**Table 1:** Primer sequences.

Gene	Forward 5'	Reverse
AGAP009551	CACGTCCTCGCTTCATCAAGAC	GGTTGCGCCGAATGTGTATG
AGAP011615	GAACCCGGTTGTGCTATCCT	GCACTGGTACTGCGATTCTTG
S7	GTGCGCGAGTTGGAGAAGA	ATCGGTTTGGGCAGAATGC

### 4. Degree-days Calculation

**Figure 1:**
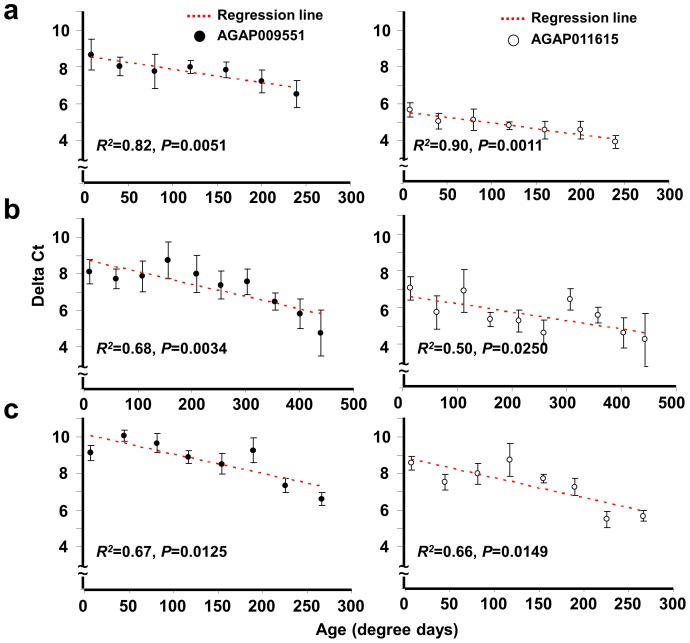
Age dependent transcript profiles in three different *Anopheles gambiae* populations. Each dot (• or ○) represents the average Δ-Ct value ± stand error at a defined age category in “degree days” for AGAP009551 or AGAP011615. The fitted regression line (red dashed-line) and regression coefficient are provided for each gene and each mosquito population. **a**) G3 strain, insectary-regulated conditions, *n = *48; **b**) Mbita strain, unregulated field laboratory conditions, *n = *58; and **c**) Mbita x Kisumu strain, MalariaSphere semi-natural conditions, *n = *85.

Degree-days are a tool that can be used in the assessment and analysis of the threshold and maximum temperatures for development of an insect. One degree day means when the average temperature for a day is one degree over the threshold temperature of growth and development. The degree day method allows an aging rate to be scaled correctly to the physiology that drives ectotherm development [26]. We monitored the temperatures during each experiment and subsequently calculated the degree-days, a metric more suitable than chronological age for grading mosquitoes. Significant correlations between Δ-Ct values and mosquito age by degree-days were determined (Fig. 1), supporting the conclusion that the transcriptional profiles of these two genes can be used to evaluate mosquito ages in laboratory or natural conditions. A model of age-related gene expression levels was constructed using a robust regression analysis. The transcriptional profile on gene AGAP009551 and AGAP011615 from three mosquito populations were used (n = 362). By constructing a probability distribution of age at which gene expression falls below a critical threshold and fitting this to age data using over dispersion parameter estimated by Maximum Likelihood. The results from the analyses provided strong support for adequacy of the age-related consistency reliability for two target genes in three mosquito populations. The probability of goodness of fit statistic are all smaller than 0.0001.

### 5. Statistical Analysis

#### Total effective temperatures

Mosquitoes and *Plasmodium* parasites in the vector stage require certain thermal accumulation, designated the total effective temperatures [27], to reach to a particular developmental stage. The accumulated degree-days, defined as the accumulated product of time and temperature between the developmental thresholds for each day, were used to measure mosquito age instead of chronological age to reduce the impact of variable ambient temperatures that the mosquitoes may have experienced. The models for mosquito age grading based on degree days would be more generally applicable than one based on chronological age. The accumulated degree-days were calculated using the sine-wave curve method [27] with the minimum threshold temperature for *An. gambiae* development set at 18°C [28]. HOBO data loggers (Onset Computer Corporation, Pocasset, MA) were placed inside each experimental condition to monitor hourly temperature changes (see **Figure** **S1**). The averages of hourly temperatures for a day were used to calculate the degree days.

#### Multiple linear regression model for mosquito age prediction

We first examined the linear regression between transcript abundance of each gene and mosquito age (measured in degree-days and chronological days) for mosquitoes in cage studies in an insectary regulated for temperature and humidity, an insectary in which temperature and humidity were not regulated, and in the semi-natural MalariaSphere. Highly significant correlations in gene expression during aging were found in all populations. In order to test the rate of change of expression profile as a function of changes in the age, the slopes of the regression lines were examined across different population. We noticed that the same regression coefficients (*P = *0.093 and *P = *0.1123 for AGAP009551 and AGAP011615 respectively) in these three populations and this result allowing multiple linear regressions to be conducted for the three populations combined to generate a general model to predict mosquito age (in degree-days) using the transcript abundance of the two genes as independent variables. This model was validated using the mark-release-recapture method and then used to predict the age of field-collected mosquitoes from Mbita Point and Kisian in western Kenya. A non-parametric Wilcoxon test was conducted to determine the statistical difference in the detected mosquito age between parous and nulliparous mosquitoes (Z = 4.85, *P*<0.0001). All statistics were performed using JMP Genomics ver. 5.0 implemented in JMP ver. 9.0. (SAS Institute Inc.).

### 6. Informed Consent and Ethical Clearance

The animal usage protocol was approved by The University of California Irvine Institutional Animal Care and Use Committee (IACUC) (UCI Animal Use Protocol number: 2008–2774), which oversees and provides veterinary care for animal care facilities at UCI. UCI's animal care and use program is regulated by both the U.S. Department of Agriculture and the U.S. Public Health Service. In addition, UCI's animal care and use program is fully accredited by the Association for the Assessment and Accreditation of Laboratory Animal Care, an independent, international organization that inspects and evaluates animal research programs and facilities. Ethical clearance was also given by the Institutional Review Boards (IRB) of the International Centre of Insect Physiology and Ecology, Kenya and the University of California, Irvine, USA. Written consent was obtained from all volunteers. We have the owners’ permissions to access their houses for mosquito collection in Mbita and Kisumu areas.

## Results

### 1. Aging Transcriptional Profiles from Three Different Strains in *Anopheles gambiae*

Two genes, AGAP009551 and AGAP011615, identified from our previous investigation of genome-wide changes in gene expression during aging [9] were selected due to their monotonic age-dependent expression in laboratory *An. gambiae* colonies. Equivalent amounts of substrate RNA were used to ensure uniform cDNA yields and the threshold cycle (Ct) values for the reference gene (S7) across all samples were made for all gene amplification reactions. Measurements of AGAP009551 and AGAP011615 transcript levels were acquired for three independent *An. gambiae* populations (laboratory, Kenya cage and MalariaSphere) at several ages (Fig. 1). AGAP009551 and AGAP011615 increased in abundance with aging (lowering Δ-Ct values) for all three populations. Mosquito aging rate is expected to be proportional to the time spent at a given temperature. It is worthy noting that the slopes of the calculated regression lines were not different among the three populations (*P = *0.093 and *P = *0.1123 for AGAP009551 and AGAP011615 respectively), supporting the conclusion that the rate of transcription accumulation per degree day was equivalent across the three populations with different genetic background and ambient temperatures. The correlation between transcript abundance and mosquito age measured by chronological days also was same to that measured by degree days (see **Figure S2**). The daily temperature cycles may have been fairly stable (Figure S1) during the experiments and may have contributed to the similarity of chronological and degree-day results.

### 2. Mark, Release and Recapture (MRR)

A multiple linear regression method that models the relationship between two or more explanatory variables and a response variable by fitting a linear equation was used to construct a model to predict mosquito age based on transcript profiles of the two genes (R^2^ = 0.65, *P*<0.0001) in three mosquito populations (G3, Mbita and Kisumu X Mbita ). The model is:
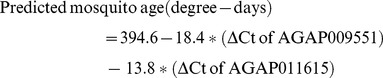
(1)


**Figure 2:**
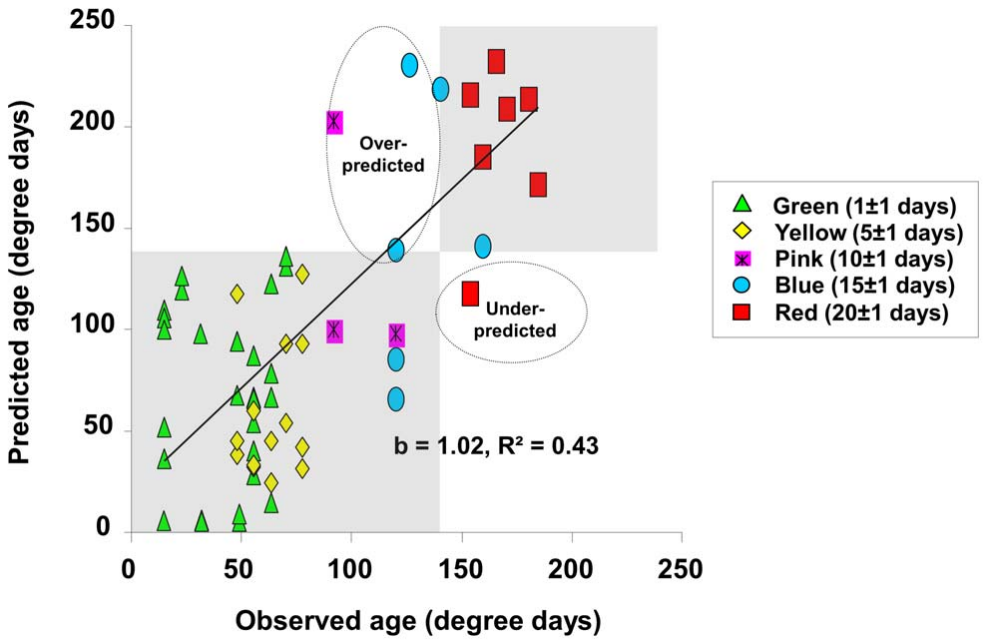
Correlation between the age of released *Anopheles gambiae* mosquitoes and that predicted based on the transcription profiles of two genes. Fluorescently labeled, released and re-captured (MRR) mosquitoes (*n = *63) of known age were used in this experiment. The data points outside of the shaded areas in the figure represent mosquitoes whose age was over-predicted (3) or under-predicted (1) by the molecular markers. The b represented the slope of regression line. The number of each age group mosquitoes was used to age prediction: Green = 32, Yellow = 15, Pink = 3, Blue = 6 and Red = 7.

The estimated parameters were listed in Figure S3. We used the MRR method to determine whether the above age-grading model is a good predictor of mosquito ages. MRR was conducted in self-contained large MalariaSphere environment. The Malariasphere is located in malaria-endemic western Kenya and therefore no mosquitoes were released into the environment. Five different colors of fluorescent powders were used to label female mosquitoes (green, yellow, pink, blue and dark-red fluorescent powders for age groups 1, 5, 10, 15 and 20 days post emergence, respectively). A total of 3,000 marked mosquitoes, 600 for each age category, were released into the MalariaSphere in April 2010 and allowed to engage in natural behaviors such as mating, oviposition and human blood feeding. Released mosquitoes were recaptured for eight successive days following release by aspirating individuals resting in the huts in the MalariaSphere. Mild weather conditions prevailed during the experiment with hourly temperatures ranging between 21.7 and 28.5°C. The daily temperature changed was recorded by HOBO data loggers in both Kenya cage condition, before released, day1 to day 28 days old, and MalariaSphere, lasting for 8 days (S1). Approximately 20 mosquitoes were recaptured each day, and a total of 151 mosquitoes among the 3,000 marked and released mosquitoes were recaptured during the 8 day post-release period. The proportion of different age groups from daily re-captured mosquitoes was showed in Figure S4. The transcript abundance of AGAP009551 and AGAP011615 from 63 recaptured mosquitoes was determined and their predicted ages calculated using the model. Analysis of the predicted ages from our model and actual ages of the released mosquitoes revealed a significant correlation (Fig. 2, R^2^ = 0.43, *P*<0.0001). Among the 63 mosquitoes tested, if 143 degree days is used as the threshold for a female mosquito to be capable of transmitting malaria [13], only 1 mosquito age was under-predicted and 3 were over-predicted (Fig. 2), yielding an error rate of 6.3%. Thus, this result demonstrated the utilities of the gene expression-based age grading method [9], [16], [17] for discriminating mosquitoes capable of transmitting malaria in western Kenya.

### 3. Apply Age-grading Molecular Markers to Field Collected Mosquitoes in West Kenya

**Figure 3:**
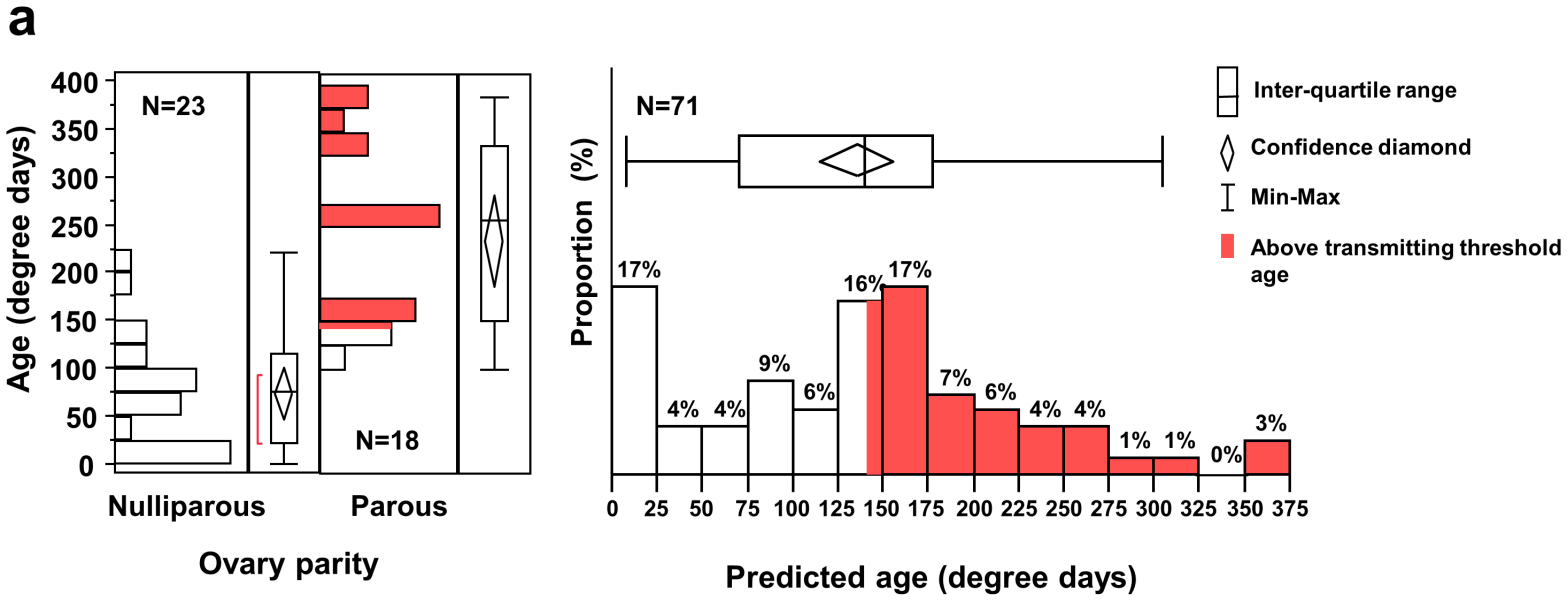
The frequency histogram and quantile plots of predicted age (in degree-days) of field- collected *Anopheles gambiae* mosquitoes. Mosquito age was determined based on the transcription profiles of the two molecular markers. a) Mosquitoes were collected from Mbita, Suba District, western Kenya, and were classified based on parity status mosquitoes. N means the number of mosquitoes collected and used for age predition; and b) Mosquitoes were collected from Kisian, Kisumu District, western Kenya. The red bar represents those mosquitoes that lived sufficiently long enough (>143 degree-days) to potentially transmit malaria.

We collected *An. gambiae* mosquitoes from two malaria endemic sites in west Kenya to validate further the molecular markers and identify the age of mosquitoes. First, mosquitoes were collected in Mbita, Suba District, in April 2010 to determine whether the ages of *An. gambiae* female mosquitoes were consistent with raw age classification based on mosquito parity. Parous mosquitoes have completed at least one gonotrophic cycle and therefore are usually >7 days post emergence whereas nulliparous mosquitoes usually have not taken a blood meal and are generally younger. The theoretical age of nulliparous and parous mosquitoes was calculated at ideal condition. In this study, we fund the relative older mosquitoes than the theoretical calculation in field mosquitoes. Transcript profiles (AGAP009551 and AGAP011615 ΔCt values) were significantly different between parous and nulliparous mosquitoes (*P = *0.0014 and *P*<0.0001, respectively). All nulliparous mosquitoes were estimated to be younger than 200 degree-days with the majority of them less than 100 degree-days, with an average 73 degree-days (95% confidence interval [CI] 47–100 degree days). As expected, parous mosquitoes were older than the nulliparous mosquitoes (nonparametric comparison using Wilcoxon method, *P*<0.0001), with an average of 235 degree-days (95% CI 187–283 degree days) (Fig. 3A). We found that the average age of field collected mosquitoes at the second study site in Kisian, Kisumu District was 124 degree-days (95% CI 109–180 degree days), with 47% of them older than 143 degree-days (Fig. 3B).

## Discussion

A genome-wide microarray screen was conducted previously for *An. gambiae* genes with transcript abundance profiles associated monotonically with aging, and a list generated of potential candidates for age grading [9], [17]. The present study extends the findings and focuses on laboratory strains and natural mosquito populations in malaria-endemic Africa. This is a major step because the age-grading biomarkers developed under laboratory conditions need to be applicable to field-derived mosquitoes with varying genetic backgrounds and ambient temperatures. Approximately 112 genes were identified whose transcripts increased or decreased monotonically with increasing chronological age and 7 candidate genes for practical age assessment were tested by quantitative gene amplification [9]. Among them, AGAP009551 and AGAP011615, were selected as age-grading biomarkers since their transcripts modulated consistently with mosquito aging at the same rates across three mosquito populations (G3 strain, Mbita strain and Mbita X Kisumu strain) and environmental conditions (regulated conditions, unregulated conditions and semi-natural conditions). These results support the conclusion that these genes have potential as excellent age-grading biomarkers among various *An. gambiae* populations in west Kenya. In the future, this method should be expanded to examine on other populations or *Anopheles* species across Africa.

In Figure 1, the significant correlations in gene expression during aging were found in all populations, but the expressional profile mosquitoes from unregulated and semi-natural conditions in Kenya showed more variation than the lab (regulated) situation. The same regression coefficients (*P = *0.093 and *P = *0.1123 for AGAP009551 and AGAP011615 respectively) in these three populations were tested. Even though we can’t ignore the relative high deviation existing in Kenya populations, we suspected the variations might be from the larvae rearing condition and other environmental factors, i.e. photoperiod [29]–[31].

AGAP009551 encodes a putative sulfotransferase associated with metabolism and the AGAP011615 product is involved in a chitin metabolic process. The function of AGAP009551 in the aging process is unknown, but changes in chitinase enzymatic activity have been associated with *D. melanogaster* aging [32], [33]. Another concern for this study was that a pyrethrum spray catch method was used for the collections from villages. Genes AGAP009551 or AGAP011615 didn’t code any protein regulate detoxification process. Genes coded to cytochrome P450 or glutathione S-transferases (GSTs) are the two big group of genes direct respond to insecticide effects [34], [35]. Genes AGAP009551 and AGAP011615 didn’t be characterized to respond to pyrethrum treatment in any published studies. Although we can not exclude the possibility of expression change to pyrethrum effect, future study should be conducted for confirmation.

The other variability of this study was the predicted age in MRR trail, especially the young groups (<7 days). The most common commercial fluorescent dust used to mark insects is Day-Glo (Day-Glo Color Corp., Cleveland, OH) and it is one of the most useful for insects with hairy surfaces [36]–[38]. We applied to the female mosquitoes by putting them in a container with a given amount of fluorescent dust and shaking the container. Small and delicate insect species can be caused immediate high mortality when placed too much fluorescent dust on the insect and it may cause undesirable side effects such as further mortality, decreased mobility, and interference with sensory organs [38], [39]. After fluorescent dust labeling of mosquitoes, we waited 24 hours of recovery time before releasing them to MalariaSphere. The gene expression profile lead to variability between predicted age and real age might be from the treatment of fluorescent dusts.

We applied the new age-grading method to determine the proportion of mosquitoes collected in the field that are sufficiently old enough to transmit malaria. The extrinsic incubation period of malaria parasites was estimated to be 111 degree-days for *P. falciparum* [13]. This estimated extrinsic incubation period reflects the duration of entire sporogonic cycle, which includes the process of fertilization of the macrogametocyte, development of the zygote and ookinete, ookinete penetration of the midgut, formation of the oocyst and subsequent sporozoite development and invasion of the salivary glands. Female mosquitoes must be old enough to ingest an infectious bloodmeal to become infected. Assuming that a female mosquito ingests its first blood meal at 32 degree-days (4 days post emergence in western Kenyan with an average ambient temperature of 26°C; [the threshold temperature for sporogonic development is 18°C]), *An. gambiae* female mosquitoes need to be at least 143 degree-days old to be capable of transmitting malaria. Using this number as the threshold age for a mosquito to potentially transmit malaria, we estimate that 47% of mosquitoes collected from Kisumu District were old enough to transmit *P. falciparum* (Fig. 3B).

The design of degree days was to provide a comparable standard to future or past studies [40], [41]. We have the stable temperature change daily and may not see the significant impact of it (Fig. 1 and Figure S2). We only used the successful MalariaSphere trails, released the same cohort mosquitoes, to this study. The successful trail defined as we collected enough number of mosquitoes at each age category (the oldest ones were at least over 3 weeks). We did fail a couple of times when no single mosquitoes was collectable over 2 weeks, especially when the thunder storm combined with severely temperature change occurred during the experiment period. It was our limitation to collect enough number of mosquitoes for age grading and have stable weather condition.

The transcription abundance of age-grading technique has valuable implications on the evaluation the effectiveness of new vector control measures that target adult mosquito survivorship, such as insecticide-impregnated bednets and life-span reducing bioinsecticides. The technique will improve the understanding of vectorial capacity and the impact of environmental changes and interventions by determining directly the age of field-collected mosquitoes.

## Supporting Information

Figure S1 The records of temperature profile to calculate degree days in three experimental conditions. The Y-axis represented the calculating degree days, temperature - developmental threshold (18°C), in hourly while the X-axis was the chronological age in days. **a**) insectary-regulated conditions, for 30 days; **b**) unregulated field laboratory conditions, for 46 days; and **c**) MalariaSphere semi-natural conditions, for 36 days.
(PDF)

Figure S2 Age dependent transcript profiles in three different *Anopheles gambiae* populations. Each dot (• or ○) represents the average Δ-Ct value ± stand error at a defined age category in “days” (chronological age) for AGAP009551 or AGAP011615. The fitted regression line (red dashed-line) and regression coefficient are provided for each gene and each mosquito population. **a**) G3 strain, insectary-regulated conditions, *n = *48; **b**) Mbita strain, unregulated natural conditions, *n = *58; and **c**) Mbita x Kisumu strain, MalariaSphere semi-natural conditions, *n = *85.
(PDF)

Figure S3 Standard errors for the coefficients in the regression model for the age-prediction regression equation.
(PDF)

Figure S4 The daily proportion of various age groups from MRR mosquitoes. Five groups (1, 5, 10, 15 and 20 days post emergence) of female *An. gambiae* mosquitoes of Mbita strain were fluorescent labeled, released and recaptured. About 20 mosquitoes were re-captured randomly each day during the 8 day post-release period. The mosquitoes were collected inside the hut, a traditional Kenyan homestead.
(PDF)
