# Supplementary figures and images for: Gene Expression-Based Biomarkers for Anopheles gambiae Age Grading
Source: PLoS One. 2013 Jul 23;8(7):e69439. doi: 10.1371/journal.pone.0069439 (PMC3720620; doi:10.1371/journal.pone.0069439)

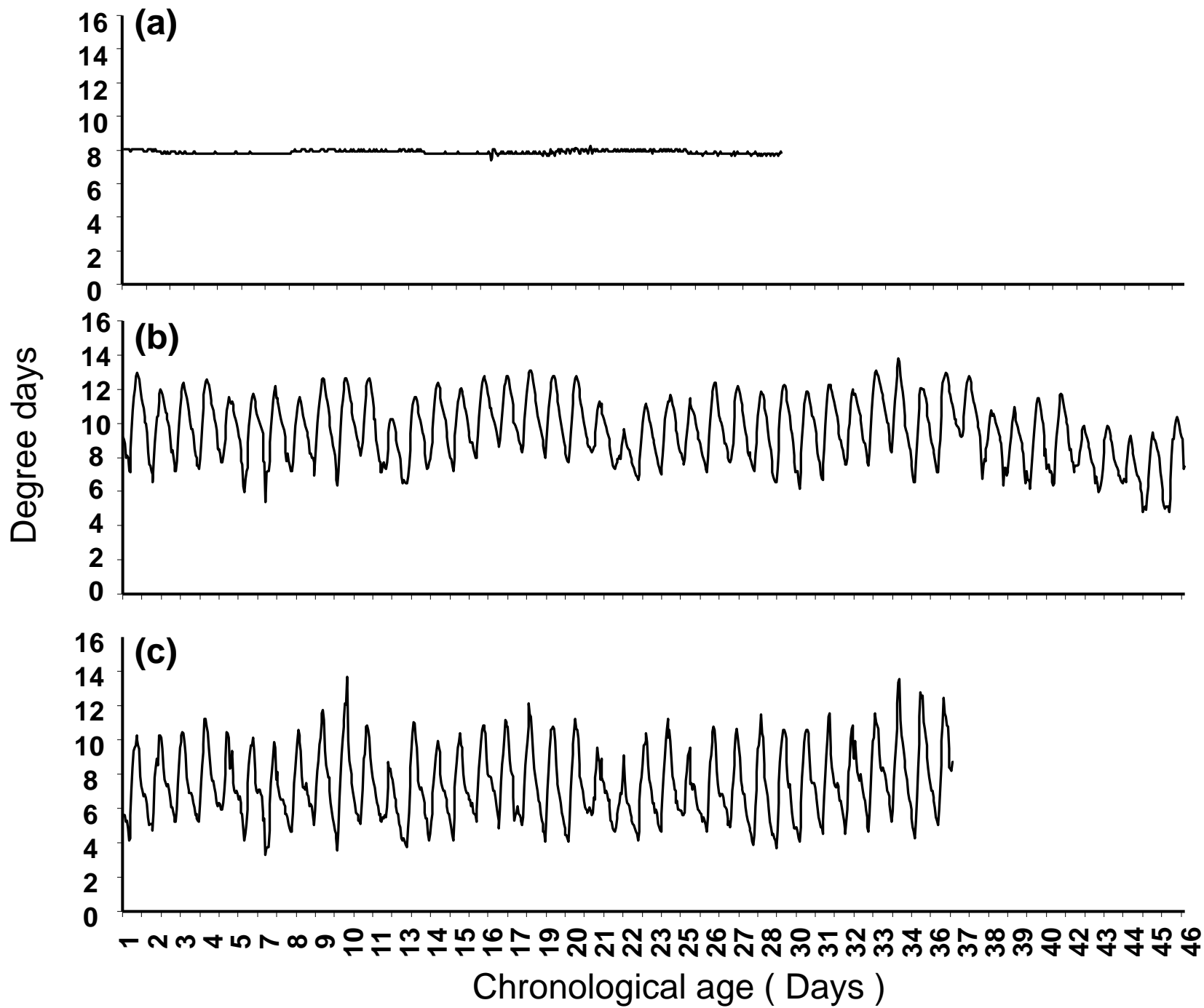

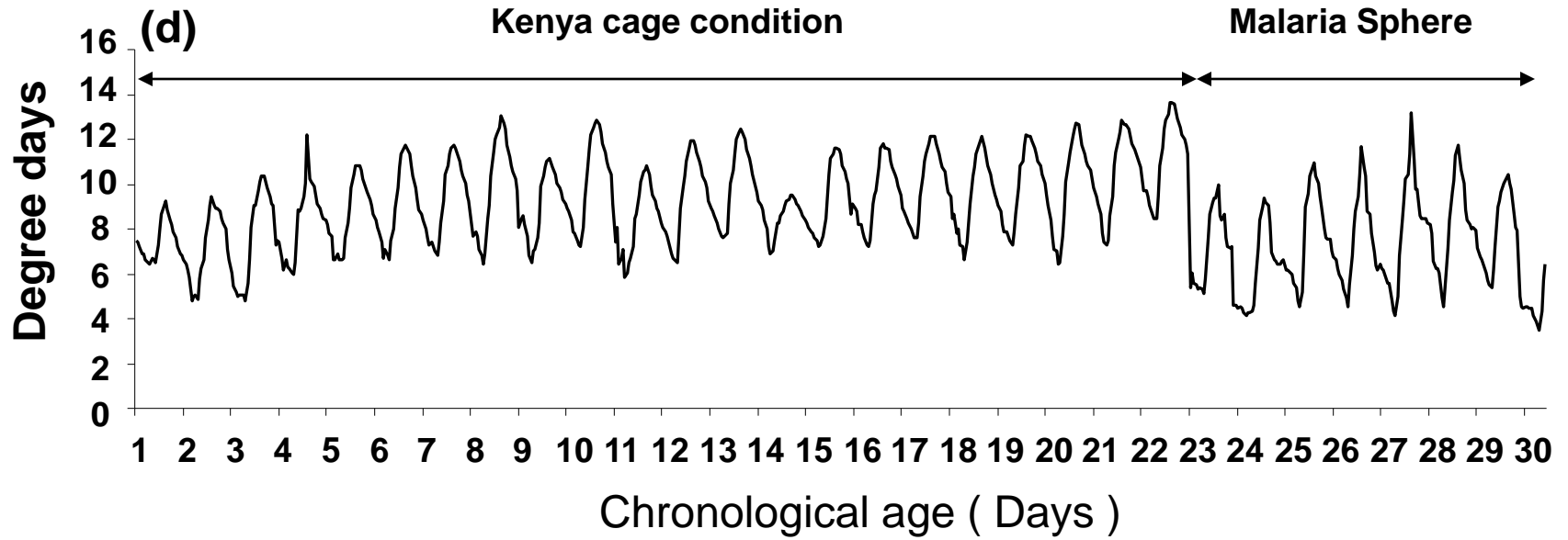

Supplement: Figure S1 — The records of temperature profile to calculate degree days in three experimental conditions. The Y-axis represented the calculating degree days, temperature - developmental threshold (18°C), in hourly while the X-axis was the chronological age in days. a) insectary-regulated conditions, for 30 days; b) unregulated field laboratory conditions, for 46 days; and c) MalariaSphere semi-natural conditions, for 36 days. (PDF) [file pone.0069439.s001.pdf]

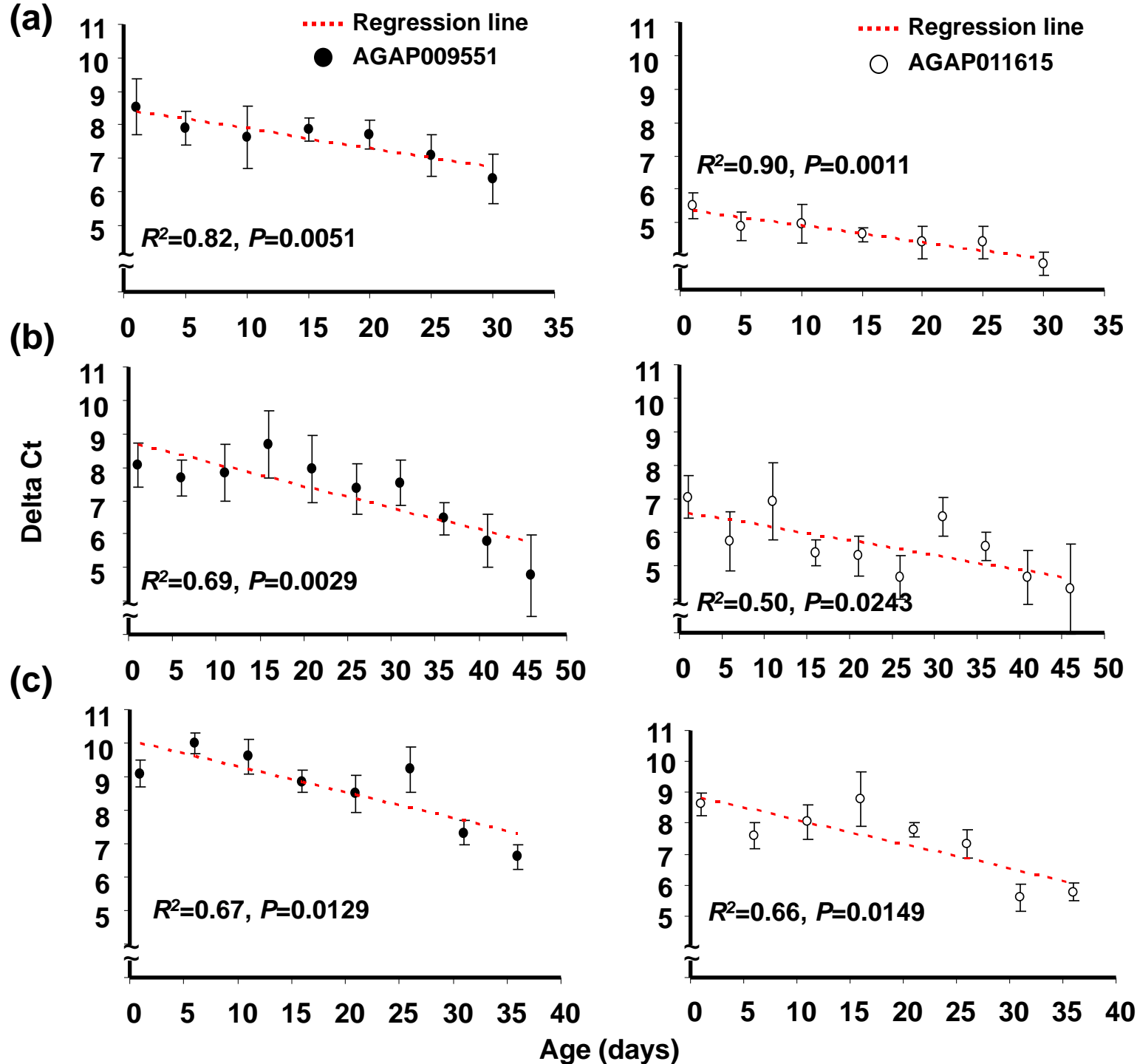

Supplement: Figure S2 — Age dependent transcript profiles in three different Anopheles gambiae populations. Each dot (• or ○) represents the average Δ-Ct value ± stand error at a defined age category in “days” (chronological age) for AGAP009551 or AGAP011615. The fitted regression line (red dashed-line) and regression coefficient are provided for each gene and each mosquito population. a) G3 strain, insectary-regulated conditions, n = 48; b) Mbita strain, unregulated natural conditions, n = 58; and c) Mbita x Kisumu strain, MalariaSphere semi-natural conditions, n = 85. (PDF) [file pone.0069439.s002.pdf]

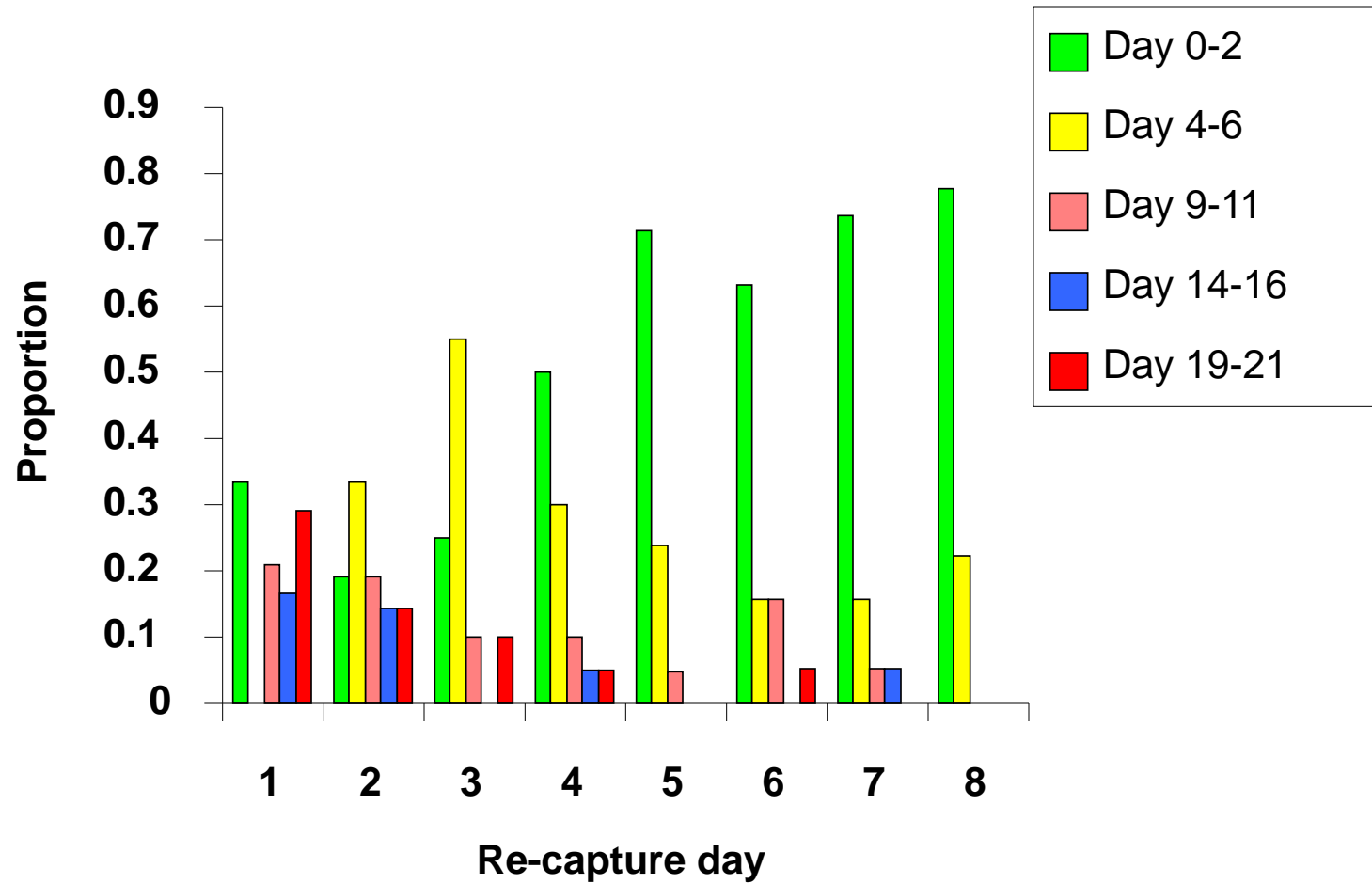

Supplement: Figure S4 — The daily proportion of various age groups from MRR mosquitoes. Five groups (1, 5, 10, 15 and 20 days post emergence) of female An. gambiae mosquitoes of Mbita strain were fluorescent labeled, released and recaptured. About 20 mosquitoes were re-captured randomly each day during the 8 day post-release period. The mosquitoes were collected inside the hut, a traditional Kenyan homestead. (PDF) [file pone.0069439.s004.pdf]
