# Supplementary material for: Gene Expression-Based Biomarkers for Anopheles gambiae Age Grading
Source: PLoS One. 2013 Jul 23;8(7):e69439. doi: 10.1371/journal.pone.0069439 (PMC3720620; doi:10.1371/journal.pone.0069439)
Supplement: Figure S3 — Standard errors for the coefficients in the regression model for the age-prediction regression equation. (PDF) [file pone.0069439.s003.pdf]

S3. Estimated parameters in the regression model for age prediction

Term	Estimate	Std Error	t Ratio	Prob>|t|
Intercept	394.6	43.9	8.86	<.0001*
AGAP009551	-18.4	4.7	-3.88	0.0002*
AGAP011615	-13.8	3.9	-3.48	0.0008*
